# Morphological features of magnetorheological elastomer degradation under a natural weathering environment

**DOI:** 10.1038/s41598-024-51736-x

**Published:** 2024-01-12

**Authors:** Mohd Aidy Faizal Johari, Saiful Amri Mazlan, Siti Aishah Abdul Aziz, Nursyafiqah Zaini, Nur Azmah Nordin, Ubaidillah Ubaidillah, Ramesh V. Upadhyay, Shahir Mohd Yusuf

**Affiliations:** 1 Engineering Materials & Structures (eMast) Ikohza, Malaysia-Japan International Institute of Technology (MJIIT), Universiti Teknologi Malaysia, 54100 Kuala Lumpur, Malaysia; 2 Department of Mechanical Engineering, College of Engineering, University of Business and Technology (UBT), P.O. Box No 21448, Jeddah, Saudi Arabia; 3 Faculty of Applied Sciences, Universiti Teknologi MARA Cawangan Pahang, 26400 Bandar Tun Abdul Razak Jengka, Pahang Malaysia; 4 Mechanical Engineering Department, Faculty of Engineering, Universitas Sebelas Maret, J1. Ir. Sutami 36A, Ketingan, Surakarta, 57126 Central Java Indonesia; 5 Dr K C Patel Research and Development Centre, Charotar University of Science and Technology, CHARUSAT Campus, Changa, 388421 Gujarat India

**Keywords:** Polymers, Atomic force microscopy, Scanning electron microscopy

## Abstract

It is well known in the field of materials science that a substance’s longevity is significantly influenced by its environment. Everything begins with the initial contact on a material’s surface. This influence will then deteriorate and have an extended negative impact on the strength of the material. In this study, the effect of natural weathering in tropical climates on magnetorheological elastomer (MRE) was investigated through microstructural evaluation to understand the aging behavior of the environmentally exposed MRE. To understand and elucidate the process, MREs made of silicone rubber and 70 wt% micron-sized carbonyl iron particles were prepared and exposed to the natural weathering of a tropical climate for 90 days. The MRE samples were then mechanically tensile tested, which revealed that Young’s modulus increased, while elongation at break decreased. Surface degradation due to weathering was suspected to be the primary cause of this condition. Using scanning electron microscopy (SEM), the degradation of MRE was investigated as a function of morphological evidence. Upon examination through SEM, it was noted that the weathering effects on the morphology of the exposed samples showed distinct characteristics on the degraded surfaces of the MRE, including numerous microvoids, cavities, and microcracks. While these features were not prominent for the MRE itself, they bear resemblance to the effects observed in similar materials like rubber and elastomer. An atomic force microscope (AFM) is used to investigate the surface topography and local degradation conditions. This observation revealed a distinctive degradation characteristic of the MRE in connection to natural weathering in tropical climates. The surface damage of the MRE samples became severe and inhomogeneous during the environmental aging process, and degradation began from the exposed MRE surface, causing the mechanical characteristics of the MRE to significantly change.

## Introduction

Natural weathering is a natural force that shapes the environment, causing the surrounding materials to degrade. The degradation process involves a few factors dependent on natural sources, such as sunlight, wind, rain, and many more. Nature’s factors attacked the surface of the materials gradually and severely over time. As a result of these microscale processes, the properties of the materials deteriorated. However, little is known about the type of microscale degradation and how it affects materials, particularly in real-world applications exposed to this challenging environment. Magnetorheological elastomer (MRE), an advanced and smart material poised to meet future technological demands, is compelled to confront the challenges of this unpredictable environment regardless of its readiness. Furthermore, MRE is believed to be capable of being used in environmentally exposed devices such sensors, structural components, and even specialized applications like adaptive camouflage systems, environmental monitoring sensors, and structural health monitoring systems, where MRE are intentionally exposed on surfaces to dynamically respond to environmental conditions for effective concealment.

In the beginning, Grassie et al.^1^ in 1965 on polymer degradation commenced with a very specific focus on physicochemical properties. However, it was not until Torikai et al.^2^ started a continuous study on environmental degradation 30 years later. Investigation of environmental effects essentially started with an emphasis on how polymers behaved in response to environmental factors, also known as photooxidation degradation. Later, Torikai and Hasegawa^3^ presented an accelerated photodegradation study 10 years later to commemorate the study’s foundation. Several works^4,5^ were conducted at the same time on the impact of temperature and other radiation-induced degradation of polymers, but the clarification was confined to chemical properties. In contrast, Ogawa et al.^6^ added mechanical parameters into their investigation by taking elongation at break and tensile strength into account. However, there is still no investigation on this material degradation that focuses on the early influence of the material’s surface that promotes the aging process and eventually degrades the material.

The knowledge acquired from the early work was then applied to more advanced materials such as elastomers and rubber-based composites. Many scientific initiatives^6–10^ have been conducted to investigate the degradation of these materials. The environmental conditions have become more precise, involving natural weathering^11–15^ rather than just at the laboratory scale or under controlled accelerated conditions. Extreme weathering conditions or climate fluctuations may have left the data with insufficient information to understand and accurately depict the early-stage conditions under natural weathering impacts. Natural weathering factors such as ultraviolet (UV), ozone, wind, rainwater, and a few other effects all play a vital role in material degradation, and the process is entirely controlled by nature. Studies on the UV effect^7,16–19^ on polymer and composite rubber found that UV irradiation altered the surface’s mechanical characteristics, increasing stiffness while decreasing elongation at break and tensile strength. As a consequence, the rubber hardened and became brittle. It should be noted, however, that UV irradiation is believed to only harm the surface that receives direct sunlight. In addition, the systematic investigation carried out did not pay close attention to the specific microscale properties of the surface. This implies that a surface or morphological study is needed to determine the initial damage of a material and the manifestation of the damage, which may be quite specific to this UV irradiation.

Another major component that has a considerable impact on the material during weathering conditions is ozone attack. Ozone is a harmful air pollutant comprising three oxygen atoms that strongly react with rubbery materials at ground level. The ozone radical breaks the polymer link, and the ozone attack showed fissures on the material’s surface, leading to the greatest failure. This ozonolysis is also known as ozone cracking in polymers. Few studies^20–24^ have been conducted and established an understanding of the ozone effects on elastomeric and rubber composite materials. The investigations concluded that the changes in material properties were caused by ozone aging. The creation of cracks on the surface of the materials was also discovered; however, the cracks formed were small and discontinuous. Nevertheless, these experiments were not carried out over a long enough time to detect more changes in the cracks as a result of ozone influence. It has been reported^12^ that over time, a succession of cracks perpendicular to the applied stress emerges. When these cracked surfaces are subjected to more ozone, the crack widens and deepens, resulting in surface cracking that not only detracts from aesthetics but can also shorten service life. Moreover, most of the studies described thus far were conducted in the laboratory, accelerated, and significantly within a short exposure period.

In regard to environmental factors, UV and ozone are not the only factors that impact the degradation process. It is primarily controlled by microscale degradation processes and modifications impacting molecule chains, particularly cross-linking and chain scission. Few researchers^4,18,25^ have examined and discovered that cross-linking reactions hardened the materials, whereas chain scission softened them. There are a few more factors; however, they are highly dependent on weather and climate conditions. Previously, studies on the environment’s temperature, humidity, precipitation (rainwater), and the effect of wind speed were conducted^12,14,15,26^, including accelerated weather studies^27–29^ to understand the contribution of all of these variables to the degradation process. All of the variables were discovered to serve a vital impact in helping the deterioration process. In the harsh environment, the degraded surface is worn and then naturally leaches away by the erosion process, exposing a fresh layer that is then reattacked by UV radiation and ozonation. This continuous process is responsible for the overall durability performance under a weathering environment. The degradation of the materials varies depending on the climate, and it will be advantageous to lay the framework for competent and reliable data collection. To date, climate classification for the degradation of materials is in its infancy^30^.

To date, research on the environmental effects of MRE is very limited, let alone studies on the microscale effects. The majority of research has been done on materials that are comparable to MRE, namely, rubber and elastomer materials, including composites^31^. However, these studies are almost exclusively focused on changes in mechanical and chemical properties. Worryingly, the degradation process involving natural weathering may interfere with the mechanical properties over time, further undermining the material’s durability. Changes in the mechanical properties of the material can then be recognized, but it is uncertain whether, where the effect begins on a microscale that cannot be seen with the naked eyes. As a consequence, it will be advantageous if early changes at this microscale can be identified and precautionary measures adopted to avoid worse failures.

Researchers have numerous alternatives for observing changes, and this capability is well recognized in scanning electron microscopy (SEM), which is one of the most fascinating examination methods and has provided the groundwork for most of what we know about micro- and nanoscience. However, due to the limits of SEM in observing the sample surface only on magnification in two dimensions (*x* and *y*), another alternative instrument is atomic force microscopy (AFM), which can provide magnification in three dimensions (*x*, *y*, and *z*). This is significant because researchers may directly estimate the height of a sample feature from an AFM image, and in this case, the height of the degraded layer on the MRE surface can be measured. Unfortunately, the use of AFM in degradation investigations recorded in the literature is limited^30,32,33^. There have been no systematic studies on the use of AFM to verify degradation at the microscopic level in weathering conditions. The current study attempts to assess the influence of a few factors from natural sources that contribute to MRE degradation in a natural weathering environment. With a 90-day exposure period, special consideration is paid to the morphological characteristics and, to a lesser extent, the tensile qualities of MRE. The study also investigates the nature of surface deterioration using SEM as well as AFM as a new means of evaluating deteriorated surfaces.

## Results and discussion

### Morphology of unexposed samples

**Figure 1:**
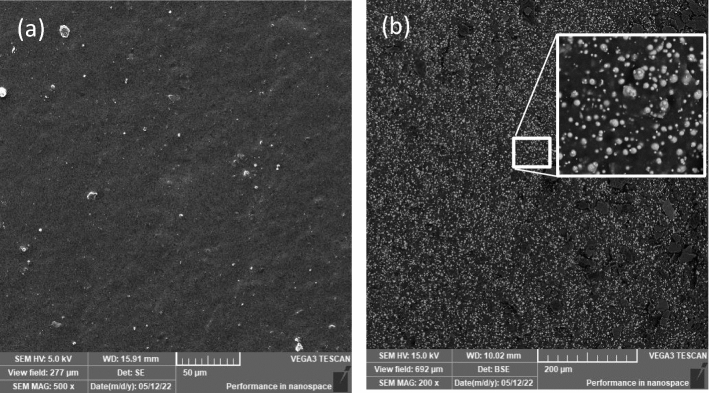
SEM micrographs of (**a**) the external side surface of the MRE sample and (**b**) the MRE longitudinal section details.

The sample was verified for flaws during production and sample preparation before being exposed to weathering conditions. At the same time, the original state was recorded for comparison with the exposed samples. Figure 1 shows a surface and cross-section SEM micrograph of the MRE sample, which is used as a qualitative reference and for comparison with the exposed sample. The original samples are discovered to have uniform flat surfaces, as shown in Fig. 1a, and similar longitudinal section surfaces, as shown in Fig. 1b. The CIPs in the MRE were spread uniformly and isometrically. There were indications of voids and aggregation within the CIP, although only in a modest proportion.

**Figure 2:**
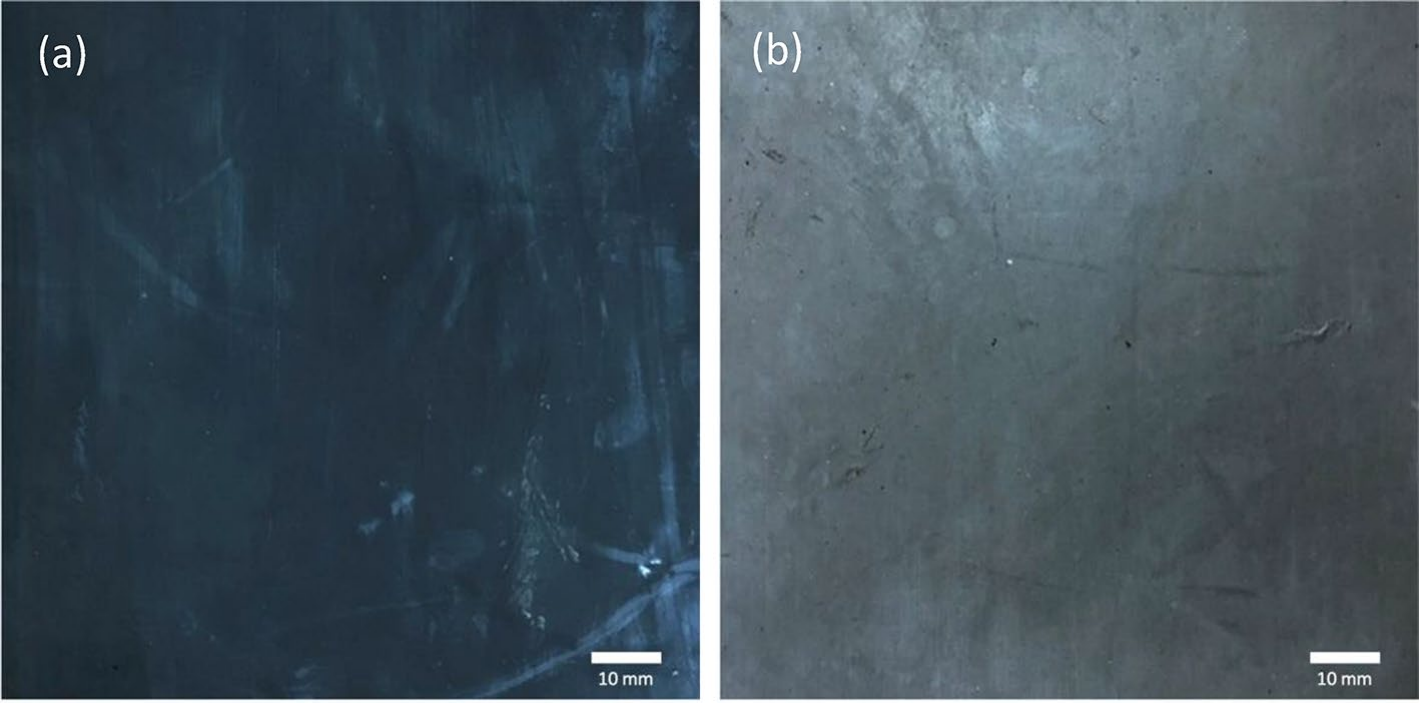
The color of the MRE surface (**a**) before exposure and (**b**) after 90 days of natural weathering exposure.

Another sign of a good reference before exposure is a bright and sharp coloration without being pale or fading. The MRE sample used in this study was a uniform dark blackish color. The discoloration is a defect that occurs as a result of prolonged exposure to UV radiation and is a sign of surface degradation. This is typically linked to photooxidation because the development of color occurs concurrently with other signs of photooxidation in the overall aging process^1^. As a result, as illustrated in Fig. 2, any defect caused by discoloration can be easily identified and detected. As shown in Fig. 2a, as the exposure time increased, the surface of the MRE sample faded from bright black to dull gray. This is an early indication of surface discoloration caused by MRE surface degradation. However, scientific evidence and evaluation of the fault, as well as a few other degradation elements, will be necessary to offer records and patterns of the defect’s deterioration process. The condition of the unexposed surface is extremely important because it depicts the original source when evaluating changes in the surface morphology of the MRE. It is understood that the process of MRE degradation under weathering influence begins only with the surface exterior.

### Observations during exposure

**Figure 3:**
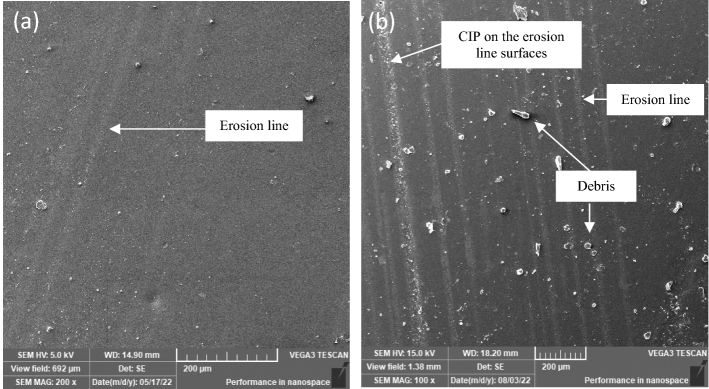
SEM image of surfaces for MRE (**a**) after 30 days^15^ and (**b**) after 90 days of natural weathering exposure.

The hypothesis that the MRE sample gradually degraded after prolonged exposure of 30 days to natural weathering was confirmed^15^. In the current study, samples were further exposed to a natural tropical climate for up to 90 days. Initial visual inspection revealed that the MRE experienced discernible discoloration during the weathering process up to 90 days, which was not evident at 30 days. As the exposure time increased, the sample surface became increasingly fuzzier. Long-term UV radiation and rainwater flow are believed to contribute to degradation with discoloration effects. The change, however, occurred on the sample’s surface and was imperceptible, which needed further scientific investigation. Therefore, detailed observation under SEM revealed more evidence of the changes. Figure 3a depicts the surface of the MRE after 30 days of exposure^15^, with the degradation process advancing to the state depicted in Fig. 3b at 90 days. At this point, the number of apparent erosion lines on the sample has expanded and grown in breadth size, with significant thickness. It is worth noting that there was clear evidence of contaminants and debris presumed to have come from both dry (wind) and wet (rainwater) environments. Some constituents of rainwater are of local origin, while others have been transported by winds from elsewhere. Mineral and organic dust are precipitated by rainwater.

**Figure 4:**
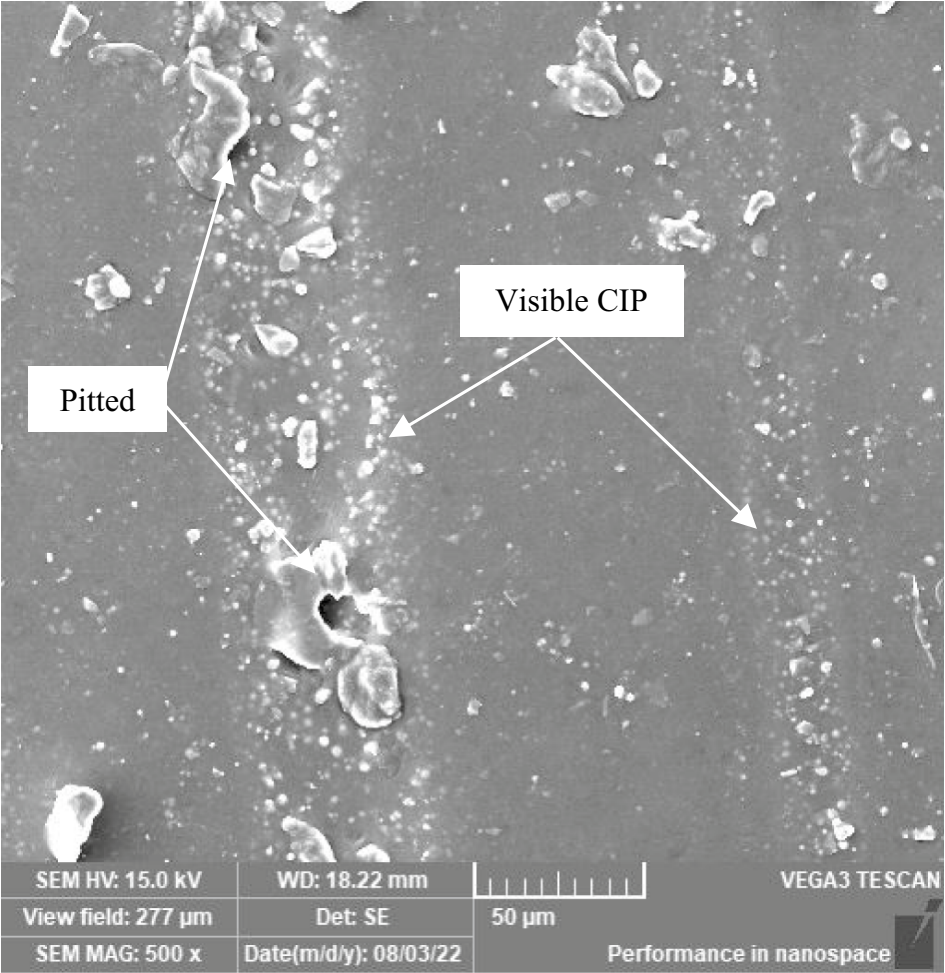
Erosion line on the MRE surface after 90 days with visible CIPs.

Contaminations in the environment that have an adverse effect on the MRE surface were induced by winds and rainwater. Rainwater contains dissolved gases that affect pH values of approximately 5.5 or lower, whereas the presence of chlorides in rainwater exacerbates the quality of erosion properties. Simultaneously, the repeating process of tropical climate involving these two parameters has aided in the precipitation of the degradation process. As the exposure time increases, the erosion line expands, and pits appear more prominently on the sample surface. During the day, UV radiation from the sun strikes the surface of the MRE, forming a thin layer on the surface that is chemically modified. Following that, rainwater leached away soluble degradation stuff during the rainy day, exposing a fresh layer that could be attacked by UV radiation once more. This repeating process has previously been observed by^18^ in different materials that experienced similar conditions. Erosion lines allowed air and rainwater to enter the matrix, causing oxidative degradation of the MRE matrix molecules. The action is suspected to be caused primarily by trapped water on the deteriorated matrix surfaces, which accelerates the process known as the photooxidation reaction and is aided by water molecules of OH^−^ and H^+^ ions. These ions primarily reflect the degree of degradation at various dominance levels. The higher the concentration of H^+^ ions in the solution is, the more acidic the solution becomes, and the solution becomes more basic as the concentration of OH^−^ ions increases. Consequently, water has a unique property that causes it to behave as an acid or a base depending on the concentration of these ions, influencing the degradation of the MRE surface. In the case of natural weathering, the process occurred naturally and solely as a result of the climate pattern. After 90 days in a tropical climate, the repeating process exposed the CIPs and pitted them, which was visible on the MRE surface, as shown in Fig. 4.

**Figure 5:**
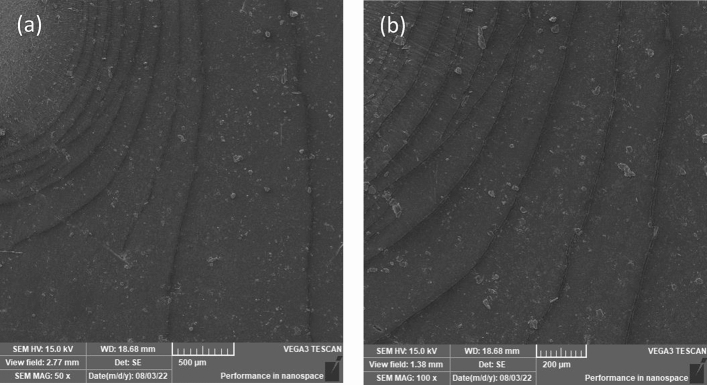
SEM images of the MRE surface with ripples and microcracking taken at various magnification fields at (**a**) 50× and (**b**) 100×.

The weathering effect on the MRE surface has been observed primarily as a result of matrix surface degradation. Furthermore, the pitted surface caused sufficient UV light scattering on the matrix surface, and after prolonged exposure to weathering conditions such as oxygen, sunlight (UV), or ozone, random cracks appeared on the surface. The material was exposed to a natural tropical climate with modest degradation, and microcracking was most likely caused by the ozone effect via chain scissions and photooxidation reactions, particularly during high humidity conditions. Further morphological investigation revealed that some surfaces had surface microcracking and a few effects rarely reported on MRE, such as ripple and the so-called elephant skin effect. Because the MRE was exposed to natural weathering conditions for an extended period of time, the ripple effect or capillary-like wave pattern observed in this study was believed to be caused by the environment’s constant back-and-forth movement of rainwater, aided by wind blowing over the surface. When the process was repeated for a longer period, especially in hot daytime conditions, the dried surface shrank, and surface tension or surface energy was created on that affected surface. Essentially, the equivalent attractive force existed between the molecules on the surface of the MRE matrix substance. As a result of the dominant surface tension, surface cracking formed, with the pattern exactly following the curvy of the ripple. Due to the sample’s hanging position outside, surface tension, gravity, rainfall inertia, and the sample’s placement all impacted the ripples, as seen in Fig. 5a, and a closer image is displayed at a higher magnification rate in Fig. 5b.

**Figure 6:**
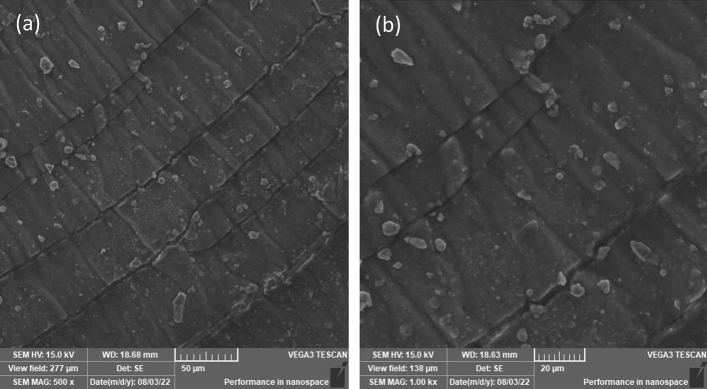
SEM images of the elephant skin effect on the MRE surface acquired across different view fields at (**a**) 500× and (**b**) 1000×.

The microcracking contour began as a smaller curved line on the affected surface and progressed over time into larger curved lines with an ovulate curve pattern. The distance between each microcracking curvy widened as it moved away from the locus point where it originated. It appears that as the ripples progressed, the surface tension or surface energy weakened, slowing down the microcracking process, and as a result, the area of the region bounded by the two curves expanded. The microcracking contour on that designated area indirectly ages the MRE sample and aids in the degradation process. A closer look at the surface near the source of the contour revealed that a crinkle pattern had formed within the ripple area. During daylight exposure, particularly in less rainy conditions, drying shrinkage characteristics were observed as a result of the natural demand for rainwater as a plasticizer, which resulted in the formation of the elephant skin effect, as shown in Fig. 6. Observations revealed that the elephant skin effect can be triggered by any drying condition that does not result in water vapor condensation on the fresh surface^34^. There is always a condition in tropical climates where sunlight hits longer before raining. In this study, there was a period of less rain and higher temperatures between the 30th and 70th days of exposure, and it was suspected that these climatic conditions influenced the formation of the elephant skin effect. A low relative humidity was also recorded during the same time period, and this condition agreed with another study^34^ in which the elephant skin effect was observed in a drying environment with low relative humidity.

The rapid drying of MRE matrix exposed surfaces via moisture evaporation and self-desiccation accelerated the formation of the elephant skin effect. Figure 6a clearly shows the elephant skin effect, while Fig. 6b demonstrates a closer image displayed at a greater magnification rate. The formation was generated by the affected skin being pulled out, resulting in wrinkles, which were also indicative of the viscoelastic behavior of the MRE that has both elastic and viscous characteristics. The viscous component that exceeded the elastic limit remained wrinkled, while the nonwrinkled segment was elastic. As a consequence, the exposed surfaces of the MRE matrix developed a rough texture and occasional cracks. Contamination from dirt and dust is one of the most evident surface conditions visible in Fig. 6. Those images are thought to be trapped by the waxy residue used as a mold release agent. The wax’s thin oily surface layer provided a foundation for dirt and dusting powders to adhere to. The stain on the surface may also be related to MRE discoloration after prolonged exposure to the environment. Further microstructural investigation was carried out to determine the mechanisms that resulted in the formation of the aforementioned defects and further degraded the MRE due to natural weathering. As a matter of consistency, it was assumed that the distribution of UV radiation, ozone, and rainwater ions was uniform across the entire MRE sample surface and felt on a flat plane. However, the defective areas observed during the study period were localized and originated from and depended on the initial conditions following the fabrication process. As a result, UV radiation, ozone, and rainwater ions have little effect on the majority of the exposed surface. Furthermore, the wind blow influenced the localized affected area, bringing all the contaminants and thus accelerating the degradation process.

**Figure 7:**
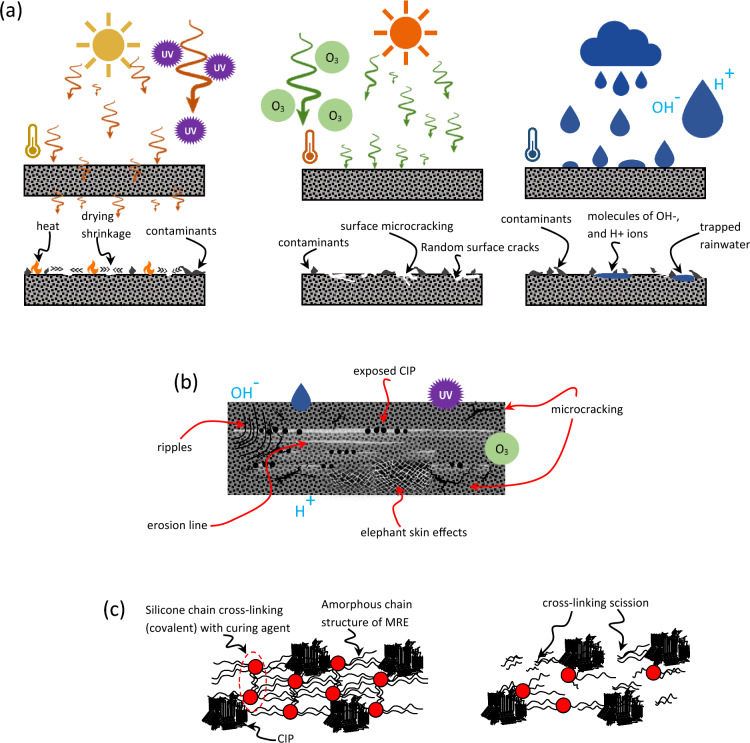
Simplified scheme of (**a**) cross-sectional view of the UV, ozone, and rainwater ion reactions and their effects on the MRE surface, (**b**) top-view of the typical 90-day effects of weathering on the MRE surface in a tropical environment, and (**c**) schematic illustration of the scission of cross-links in the MRE matrix’s molecular chain structure.

In general, the degradation process and physical changes in MRE exposed to natural weathering are highly dependent on a particular time period, so in a brief amount of time, the condition will not change permanently. MRE, on the other hand, will gradually undergo permanent changes in accordance with or directly proportional to the duration it endures for an extended period of time at an elevated factor such as temperature, humidity, wind speed and rain. As a result of the evidence gathered in this research, irreversible chemical processes occurred, implying that the MRE degraded. This undesirable progress can be simplified as illustrated in Fig. 7. Figure 7a depicts a cross-sectional representation of the reactions that occurred as a result of the influence of time, temperature, humidity, UV, ozone, and rainwater ions on the deterioration of MRE characteristics. The summation of different effects on the surface of MRE can therefore be interpreted as shown in Fig. 7b. As previously stated, the main contributor to the cracking of the MRE surface was related to cross-linkage scission caused by ozone impacts, as seen in Fig. 7c. The understanding of this process has spurred more inquiry since it is needed to comprehend the characteristic changes at a larger scale, such as mechanical properties.

### Observation of mechanical properties

**Figure 8:**
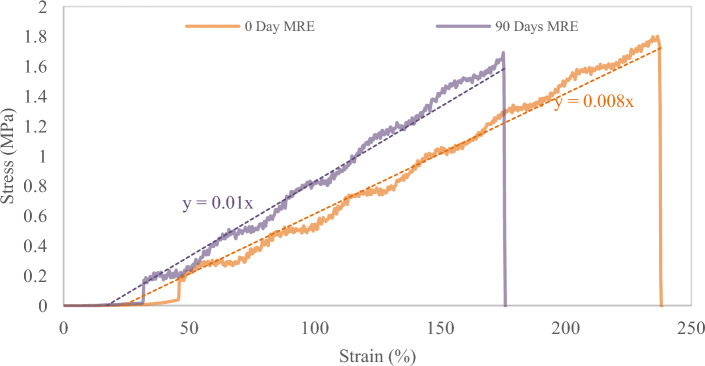
MRE stress‒strain graphs before and after 90 days of natural weathering exposure, using linear regression on the increase slope, illustrate the gradient of the elasticity section.

**Figure 9:**
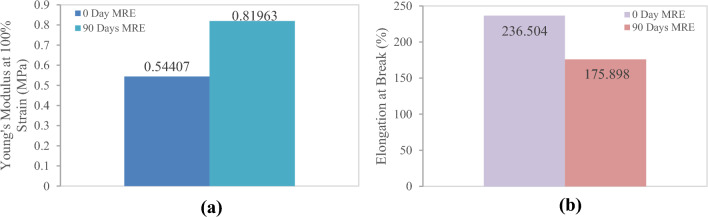
MREs (**a**) Young’s modulus and (**b**) elongation at break properties changed after 90 days of exposure to natural aging conditions.

The micrography study showed the microlevel failure that caused the MRE to degrade. It was assumed that those failures had an impact on the MRE properties as a result of the reaction process described in the preceding section. For instance, it is believed that chain scission reactions reduce the crosslink density and consequently cause the creation of more or longer dangling chain ends, softening and losing their elastic properties. On the other hand, age-related crosslinking can cause the substance to become more brittle and stiffer. The evaluation under stress and strain relation indicates that the sample after 90 days exhibits those conditions and is plotted as shown in Fig. 8. The overall pattern clearly shows that increasing the slope of the graph represents growing stiffness. The breaking point was much sooner than in the sample that had not been exposed to natural weathering, confirming the hypothesis about elasticity loss. The tensile test results also indicate that the exposed sample has a 41% difference in elastic modulus, with a 52% increase over the sample that has not been exposed to natural weathering, as depicted in Fig. 9a.

Figure 9b depicts the variance in sample elongation at break. After 90 days, the elongation at break of the exposed sample showed a clear decrease. The value was determined with a difference of at least 30% and a decrement of 26% in comparison to the unexposed sample. The degradation could be ascribed to the severity of chain scission during the 90 days of exposure with the presence of typical UV, ozone and tropical climate changes. These mechanical property vicissitudes can then be translated into variations in failure at the microstructural level, as observed under electron microscopy. The use of scientific methods and techniques in the study has demonstrated great attention to detail and a very precise failure pattern in relation to changes in the sample mechanical properties caused by the natural weathering degradation process. The image obtained showed that MRE was metamorphosed by the aging factor naturally from the tropical climate in 90 days, which has rarely been reported for MRE elsewhere. Interestingly, all of the images were particularly for tensile failure and were only compared for the sample before and after exposure.

**Figure 10:**
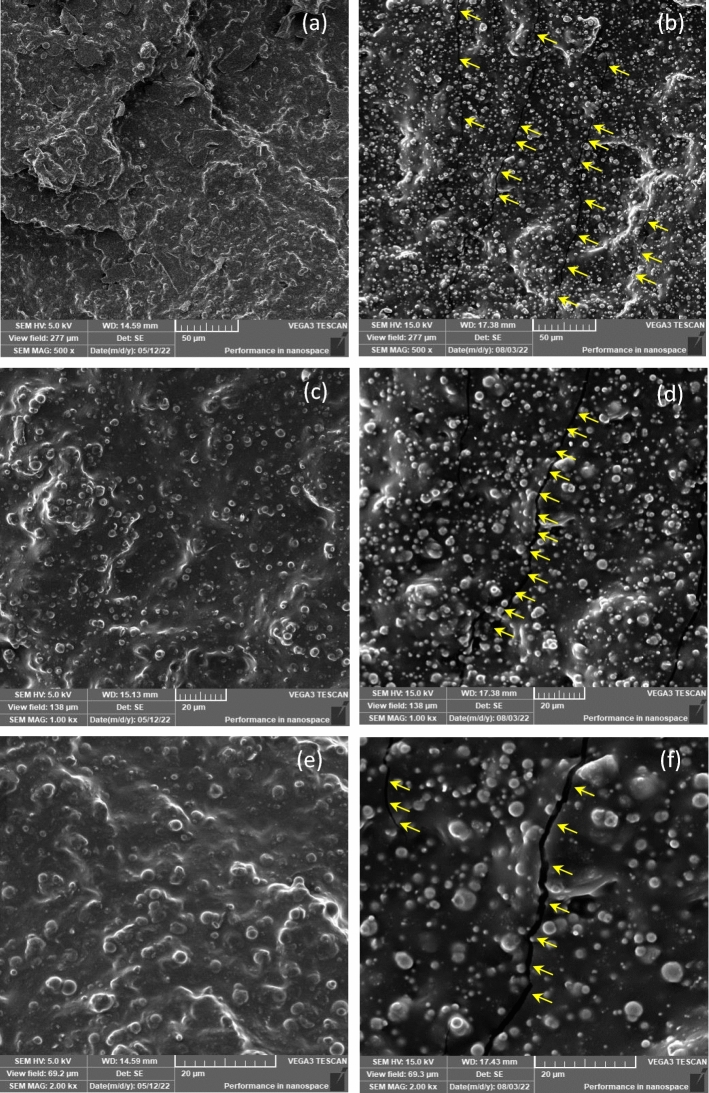
SEM images of the tensile failure surface of MRE due to natural weathering at various magnification fields: (**a**) 500× unexposed MRE, (**b**) 500× of 90 days exposed MRE, (**c**) 1000× unexposed MRE, (**d**) 1000× of 90 days exposed MRE, (**e**) 2000× unexposed MRE, and (**f**) 2000× of 90 days exposed MRE.

Even though all samples have the same matrix and particle ratio, due to exposure to natural weather for a period of time, tensile failure exhibits distinct characteristics and behavior. SEM observation reveals significantly less evidence of CIP protrusion in the unexposed sample of Fig. 10a. However, even under moderate magnification, it is clear from Fig. 10b that the exposed sample displays typical brittle failure on the matrix as well as more CIP protruding on the surfaces. Furthermore, referring to the area under the stress‒strain curve in Fig. 8, which depicts energy, reveals that the brittle failure of the exposed sample expends much less energy than the ductile failure of the unexposed sample. Ductile failure of unexposed samples occurred at higher strain and proceeded slowly, with deformation of the rubber occurring along the way, whereas brittle failure of exposed samples occurred at a much lower strain. The development of microcracks or hairline cracks on the surface was the primary distinction between the failures. In terms of brittle failure, localized residual stresses caused cracks to develop and spread more quickly in brittle media than in slightly ductile media, where the stress dispersion was delayed.

Given that UV exposure only affects the surface of the MRE matrix that is exposed to direct sunlight, UV irradiation significantly reduced the elongation at break and tensile strength of the exposed MRE and increased its stiffness. It consequently turned hard and fragile. In comparison, the SEM image of the unexposed sample at higher magnification in Fig. 10c does not appear to have any surface cracking, and the CIPs were also shown to have less bulging on the surface. While Fig. 10d depicts the surface condition, there is a crack line across the surface, as indicated by the arrows, and the surface was formed with CIP, which is clearly visible. This could be due to the firm contact with the matrix, whereas the exposed sample could have a weaker contact with the matrix, resulting in more protruded CIPs on the surface after tensile failure. Further investigation at higher magnification reveals that the crack in Fig. 10e for the unexposed MRE remains invisible; however, further cracks were observed in Fig. 10f for the MRE exposed for 90 days, indirectly justifying the hypothesis and previous findings.

### Topography evaluation by AFM

The determination of MRE surface characteristics before and after exposure was analyzed using AFM, an essential method for characterizing defected surfaces in a more localized area. The AFM contact mode method was used in this study to identify surface topography and reliably identify the differences between the conditions before and after 90 days of exposure. The AFM assessment established the durability performance of the MRE sample under natural weathering influences and its correlation with mechanical and microstructural characteristics. AFM topography evaluation of the MRE surface is thought to significantly influence and reflect the bulk properties of the MRE itself. Despite the fact that the surface scanned was localized and nanoscale in nature, the data impact of that specific area was macroscopically significant.

**Figure 11:**
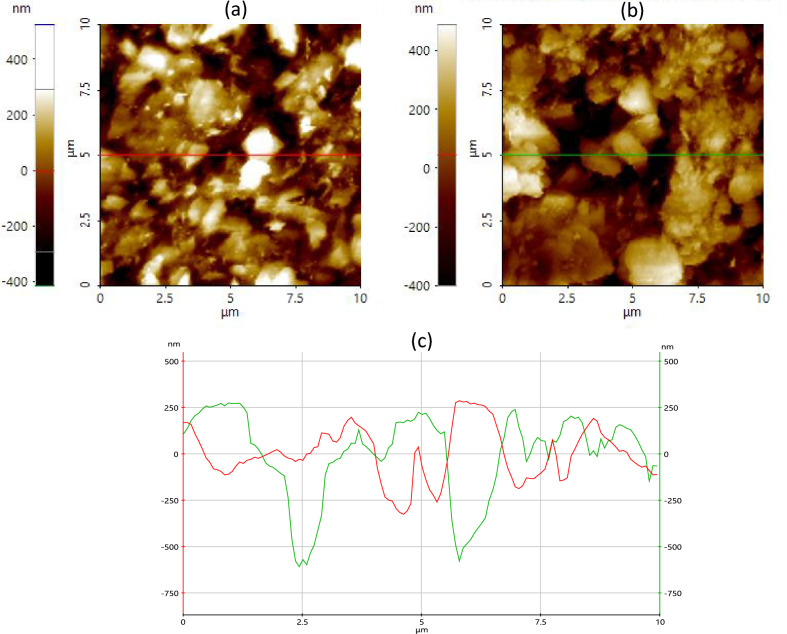
AFM images of MRE; (**a**) unexposed, and (**b**) after 90 days exposed to natural weathering. Multiline analysis of the height range for (**c**) both unexposed (red line) and exposed samples (green line).

AFM, unlike most other surface analytical methods, can investigate the topography of MRE samples down to the lowest scale under controlled conditions. Figure 11 depicts AFM images of both unexposed (Fig. 11a) and 90-day exposed (Fig. 11b) MRE samples subjected to natural weathering using contact mode. The scan size was 10 × 10 µm^2^, and the height range was 400 nm from the white to the black of the scale bar. Given that scan size has a major impact on the scaling results of AFM measurements, visual examination showed that using a large scan size (10 × 10 µm^2^) captured reasonably good images in this study. The horizontal line scan in Fig. 11c for both the unexposed (red) and exposed (green) samples indicates the height range that represents the surface condition after its reaction to natural weathering. The green line in Fig. 11c of the exposed MRE sample plainly shows a deeper value in the height range, while it is shallow for the original sample without any exposure. During natural weathering, the presence of continuous ozone concertation has higher oxidative capabilities and is noteworthy to become more aggressive at low humidity, in which ozone leads to aggressive cross-linking reactions enabling deeper cavities.

**Figure 12:**
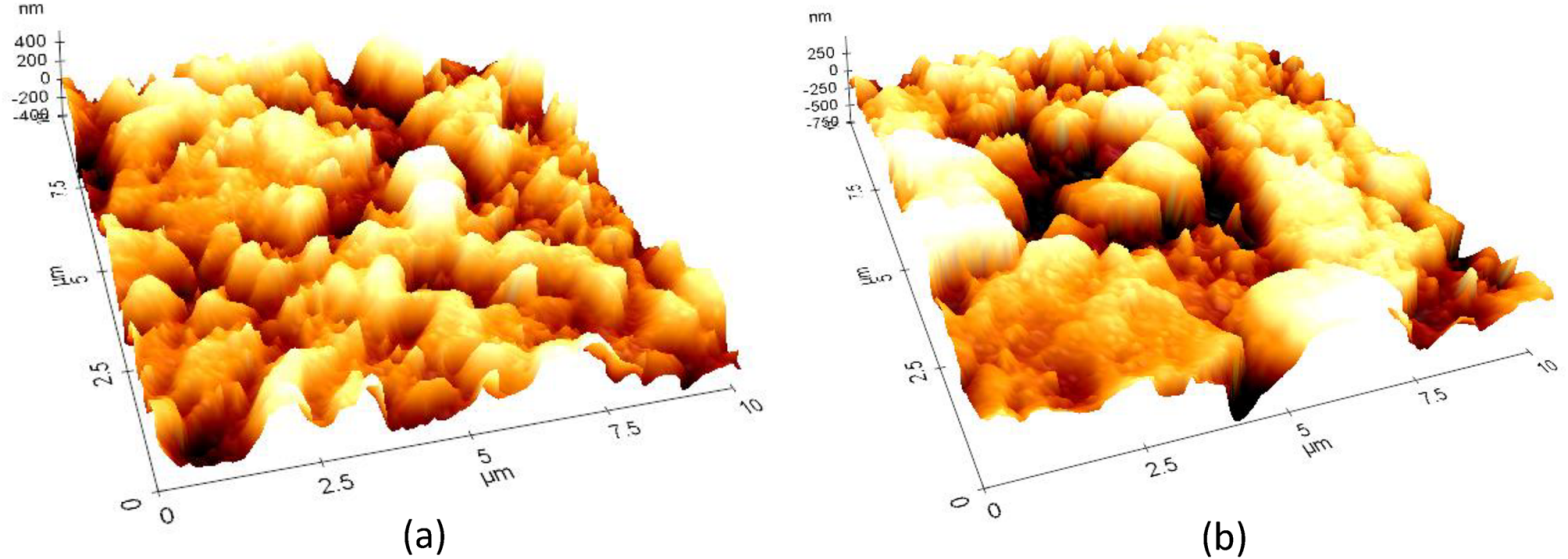
Three-dimensional AFM height range micrograph showing surface deterioration on the matrix of (**a**) unexposed MRE and (**b**) 90 days exposed MRE toward tropical weathering.

Chain scission has been observed under high humidity conditions, which results in more shallow cavities on the matrix surface. As a consequence, after 90 days of contact, the MRE sample developed cavities on the matrix surface that were much deeper than those that existed immediately after fabrication, also known as the unexposed sample. A tropical climate with a tolerable humidity percentage and constant ozone encourages cavity development. Accordingly, cavity characteristics were observed with the assistance of AFM measurements and analysis. Figure 12 depicts in greater detail the distinctive and exceptional configuration of the degraded surface of MRE caused by natural weathering. One of the peculiar features of surface degradation can be determined by the wider formation of a deep area on the MRE surface under nanoscale height range 3D projection, as shown in Fig. 12b. The cavities were consistent and optimally formed throughout the test exposure; however, in this area, the localization of the cavity was not consistent and contributed to the depth uncertainty, encouraging a similar level of uncertainty in the cavity shape. The localization of the cavity becomes evident as the exposure duration increases, resulting in the observation of a wider and deeper cavity.

## Methods

### Materials and preparation of MRE

Nippon Steel Co., Japan supplied silicone rubber in the form of a thick solution, which was used exactly as received. Soft carbonyl iron particles (CIPs) (d50 = 3.9–5.2 µm), OM grade, supplied by BASF, Germany, were mixed into a silicone rubber solution for MRE fabrication. For 10 min, the mixture was mechanically stirred at 25 °C at room temperature to ensure that the particle and silicone rubber solution were thoroughly mixed. An isotropic MRE sample with an elastomeric matrix embedded with 70% magnetizable particles was produced using a closed mold method. The curing process was carried out in a closed rectangular mold measuring 200 mm in length, 200 mm in width, and 1 mm in rectangular depth. Curing agents (0.1 wt%) were added to the mixtures and allowed to solidify for 2 h. NS625B (Nippon Steel) served as a cross-linking agent and determined the desired amorphous MRE matrix characteristics. There was no evidence of gravity segregation, and the curing pressure applied throughout the procedure was distributed evenly. The cured sample (MRE sheet) has dimensions of 150 mm in length, 150 mm in width, and 1 mm in thickness of the rectangular sinking section inside the mold. Tensile test samples were punched out using tensile cutting dies in accordance with ASTM D412, Type C standards.

### Natural weathering exposure

The natural weathering exposure of MRE samples lasted 90 days at the Malaysia-Japan International Institute of Technology, Universiti Teknologi Malaysia, Kuala Lumpur, Malaysia. Natural weathering was carried out in accordance with the established test standards. The exposed MRE sheet samples were placed in an open area facing south (3.1727, 101.7208)/(3 10′ 21.77194″ N, 10 43′ 14.8794″ E).

**Figure 13:**
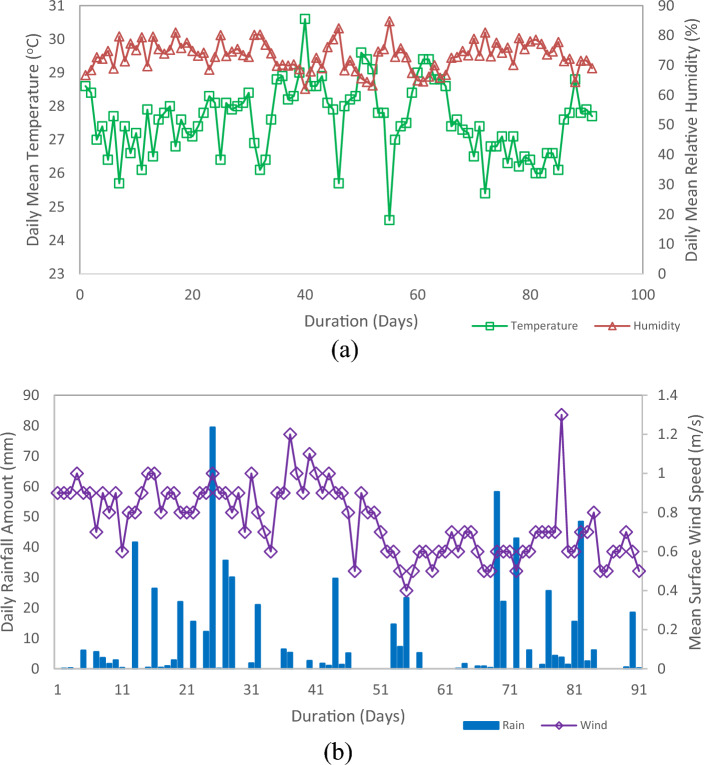
Meteorological data of (**a**) daily mean temperature and relative humidity and (**b**) daily rainfall amount and mean surface wind speed from April to June 2022.

Samples were consistently monitored throughout the process and collected after the 90 day period was completed. Before the characterization test, samples were cleaned with a cleanse cloth and left at room temperature for at least 24 h. Figure 13a,b depicts the weather conditions during the sample’s exposure to the tropical climate. The weather information was obtained from Malaysia’s meteorological department station in Kuala Lumpur, which boasts a tropical rainforest climate with consistent temperatures all year. However, there is a monsoon and rainy season from the end of the year to the beginning of the year, with the remaining months still expecting some heavy rainfall. In certain seasons, the sun is accompanied by high humidity in Kuala Lumpur. The 24-h mean temperature recorded was 27.6 °C with 56 days of rain within the exposure period. The mean relative humidity was recorded at 73% throughout the duration, and the mean wind speed readings were 0.77 m/s.

### Mechanical properties by uniaxial tensile test

The mechanical tensile properties of the MRE samples were tested before and after 90 days of natural weathering durability using a universal testing machine, Shimadzu AGX-S, Kyoto, Japan, in accordance with ASTM D 412. Dumbbell specimens were cut from the sheets using tensile cutting dies and tested in accordance with the same test standard. The tests were carried out at a controlled room temperature (25 °C) with a constant cross-head speed of 500 mm/min.

### Morphological observation

SEM was utilized to gain a better understanding of the effect of natural weathering on the MRE sample after 90 days of exposure, as well as the degradation characteristics on the surface and cross-sectional area. In this study, SEM Tescan Vega 3, Czech Republic, was used to investigate the physical aspects of the samples' unexposed and exposed areas. Before preparing the SEM sample, the exposed original MRE sheet was visually inspected for any defects or damage so that a specific location could be cut off and prepared for micrography analysis. Discoloration, surface fuzziness, and fading are examples of common degradation that can be seen with the naked eye. The identified area was then cut into segments with an approximate surface proportion of 10 mm × 10 mm for microstructure analysis. The same sample preparation procedure was used following the tensile test on the MRE sample for SEM observation, but the evaluation was confined to the area that cracked and failed during tension. The area refers to the cross-section of the fractured sample located in the gauge length area. By applying a conductive coating process to the specimen using a sputter coating machine (NS800, Novatic Scientific, Singapore), image disturbance was reduced constructively for better SEM image output. Using an auto fine sputter coater device, the surface of both samples was sputter-coated with a thin layer of gold at approximately 1 nm thickness.

The MRE sample was subsequently evaluated for topographical evaluation using AFM analysis. It was performed using Park FX40, the most advanced automatic AFM developed by Park System in Suwon, South Korea. The data obtained were mostly useful for observing three-dimensional features on the surface of the MRE sample. The surface topography of the unexposed and exposed MRE was examined within a predetermined scan region of 10 nm × 10 nm at a scan rate of 0.14 Hz. The aluminum backside-coated cantilever, with a tip length of 10–15 nm and a radius of less than 10 nm, was applied vertically to a single spot on a sample surface. Park Systems XEI was then used to create the measurement and height images.

## Conclusion

Through natural weathering durability tests, this study assessed the long-term microstructural property changes in MRE. The effects of UV, ozone and rainwater ions on the mechanical properties of the MRE were also investigated. This study aimed to better understand the degradation progress in an MRE that had experienced durability under natural weathering in a tropical climate from the beginning to the end of 90 days of exposure. UV irradiation increases the stiffness of MRE while decreasing its elongation at break, to name a few significant findings. As a consequence, the rubber hardens and becomes brittle. Morphological observations showed that this was related to surface modification, which increased the modulus by 52% while reducing the elongation performance by 26%. Extended exposure revealed more microcracks on the MRE, contributing to a significant decrease in elongation at break. UV irradiation modifies the mechanical properties of the matrix surface, whereas ozone encourages microcracking via chain scission. In addition, it has been established that a few additional elements, such as rainwater ions, humidity and tropical environment temperature, play significant roles in the MRE breakdown process. Most significantly, the degradation process in natural weathering took place only in a specific and localized area, as evidenced by SEM observations. Thus, using AFM as a novel way of measuring the features of the degraded surface and the surrounding localized region, this study investigated the nature of the degradation. This knowledge is expected to aid in evaluating MRE aging behavior and predicting lifetime, making it helpful in design and maintenance.

## Data Availability

The data presented in this study are available on request from the corresponding author.
